# Synthetic scaffolds functionalized with mesenchymal stem/stromal cells-derived extracellular matrix for bone tissue engineering: a review

**DOI:** 10.1039/d5ra02106e

**Published:** 2025-09-04

**Authors:** Margarida F. Domingues, Marta S. Carvalho, Paola Sanjuan-Alberte, João C. Silva

**Affiliations:** a Department of Bioengineering and iBB – Institute for Bioengineering and Biosciences, Instituto Superior Técnico, Universidade de Lisboa Av. Rovisco Pais Lisboa 1049-001 Portugal joao.f.da.silva@tecnico.ulisboa.pt; b Associate Laboratory i4HB-Institute for Health and Bioeconomy at Instituto Superior Técnico, Universidade de Lisboa Av. Rovisco Pais Lisboa 1049-001 Portugal; c Department of Mechanical and Aerospace Engineering and PolitoBIOMed Lab, Politecnico di Torino Corso Duca Degli Abruzzi, 24 Turin 10129 Italy

## Abstract

Bone-related injuries represent a major global challenge, particularly for the aging population. While bone has self-healing capabilities, large defects and non-union fractures often fail to completely regenerate, leading to long-term disability and the need for surgical intervention. Autologous bone grafts remain the gold standard for such procedures, but challenges such as limited donor availability and donor site comorbidity persist. Bone tissue engineering (BTE) presents an alternative approach for bone regeneration, using biomaterials, cells and growth factors that mimic the natural composition and structure of bone. Cell-derived decellularized extracellular matrix (dECM), particularly from mesenchymal stem/stromal cell (MSCs), is among the most promising biomaterials for BTE, as it closely mimics the composition of native bone ECM and provides immunomodulatory and trophic molecules that support bone regeneration. However, dECM's mechanical properties are often insufficient, requiring its combination with synthetic polymers to improve scaffold strength and structural integrity, critical to support hard tissues such as bone. This review explores the potential of MSC-dECM composite scaffolds developed for BTE, including 3D printed constructs, electrospun fibrous matrices, hydrogels and metallic scaffolds. It describes how the incorporation of MSC-dECM enhances the osteoconductive and osteoinductive properties of these scaffolds, leading to increased expression of osteogenic markers and calcium deposition *in vitro*, as well as enhanced bone formation *in vivo*. Finally, the review addresses the current challenges and future directions in advancing the application of MSC-dECM-enriched scaffolds towards clinically effective bone repair strategies, including the need to scale up MSC-dECM production, further elucidate its regenerative mechanisms, and integrate it into precise patient-tailored approaches.

## Abstract 2

This review explores the potential of MSC-dECM composite scaffolds for bone tissue engineering, including 3D printed, electrospun, hydrogel, and metallic scaffolds, and describes how MSC-dECM enhances their osteogenic and regenerative properties.

## Graphical abstract

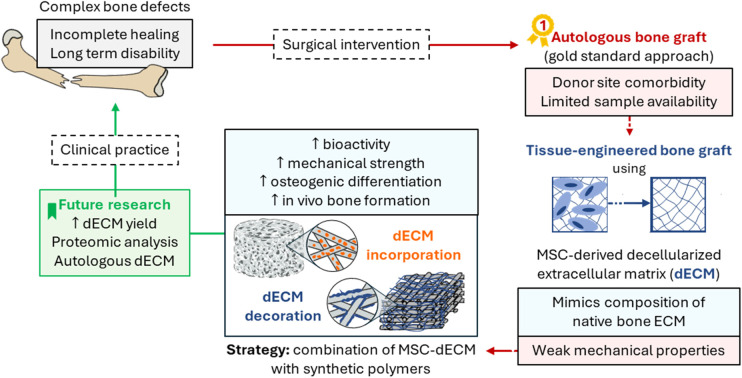

## 1. Introduction

Bone defects resulting from osteoporosis, infections, tumor resections, trauma, surgeries or congenital conditions pose a significant global healthcare burden, particularly among aging populations.^1–3^ These bone-related injuries account for nearly half of all chronic diseases in people over 50 years old,^4^ often leading to long-term disability, reduced quality of life, and the need for surgical intervention. In 2019, an estimated 178 million new bone fractures were recorded^5^ and 2–10% of these, depending on the age group, were classified as non-union fractures, which fail to heal despite the bone's self-healing capacity.^6^ Additionally, over 2 million bone-grafting surgeries are estimated globally each year.^7^ These numbers are expected to increase due to worldwide population growth and aging, which underscores the urgent need for innovative therapeutic approaches to improve bone repair and regeneration.

Bone is a highly specialized and vascularized tissue, composed of several cell types and a complex extracellular matrix (ECM), which is a dynamic network of biological molecules that provides structural support and interacts with cells by regulating physiological processes such as cell migration, proliferation and differentiation.^8^ The bone ECM consists of an organic phase (30–35%), composed of collagen type I, proteoglycans, glycoproteins, γ-carboxyglutamic acid (Gla)-containing proteins, cytokines and growth factors, which provide tensional strength, and an inorganic phase (65–70%), mainly composed of hydroxyapatite (HAp) nanocrystals, responsible for compression strength and stiffness.^9^ Within this mineralized matrix are osteoblasts, osteoclasts, osteocytes and bone lining cells.^10^ Osteoblasts synthesize new bone by secreting ECM proteins such as collagen type I, osteopontin (OPN), osteocalcin (OC) and alkaline phosphatase (ALP), while osteoclasts are responsible for bone resorption by demineralizing the matrix and degrading collagen using enzymes.^2,3^ Osteocytes, derived from osteoblasts that become embedded in the matrix during ECM secretion, act as mechanosensors and mechanotransducers, regulating the activity of osteoblasts and osteoclasts in response to biochemical and mechanical signals.^11^ Bone lining cells are inactive osteoblasts located on bone surfaces and can be reactivated by chemical and/or mechanical stimuli.^12^ Mesenchymal stem/stromal cells (MSCs), which have self-renewal capacity and the ability to differentiate into mature cell types of mesenchymal tissues (bone, cartilage and fat), are also present in the bone environment and secrete cytokines and growth factors essential for osteogenesis.^13^ Beyond its micro-scale complexity, bone also exhibits structural complexity at the macro-scale, being classified as cortical or trabecular bone and having different mechanical and functional properties. Trabecular bone, with 70–95% porosity, contains interconnected pores filled with bone marrow, whereas cortical bone, with only 5–30% porosity, forms the denser outer layer of the bone tissue, enclosing the trabecular bone.^14^ The compact structure and higher mineralization of cortical bone result in a higher Young's modulus (4.09–15.10 GPa) compared to trabecular bone (1.85–13.93 GPa), allowing it to withstand greater stress before failure.^15^ In contrast, trabecular bone, with its higher porosity, can deform more and endure higher strains before failure, playing a key role in energy absorption and load distribution.^16^

Under normal conditions, bone homeostasis is tightly regulated through a balance between osteoblast and osteoclast activity.^10^ When fractures occur, this balance is disrupted, triggering a self-repair process involving the activation of transcription factors and signaling pathways, which also involves the recruitment of MSCs to the injury site, where they differentiate into osteoblasts and secrete growth and immunomodulatory factors to aid in repair.^2^ However, complex and/or critically sized bone lesions often exceed the bone regenerative limits, for which clinical intervention is needed.^3^ Bone grafts are frequently used to address such defects and are expected to meet specific criteria, including biocompatibility, bioresorbability, osteoconductivity, osteoinductivity, mechanical strength and a structure resembling natural bone.^1,17^ Ideally, these grafts should also degrade as new bone tissue forms to replace them. Autologous bone grafts, derived from the patients, are considered the gold standard as they meet both biological and mechanical requirements,^18^ while avoiding risks associated with graft rejection and disease transmission, which can be observed with allogeneic and xenogeneic bone grafts.^4^ However, their availability is limited, and their use is associated with high donor site comorbidity.^2^

Bone tissue engineering (BTE) presents a promising alternative for bone regeneration, focusing on the reconstitution of native bone ECM by combining biodegradable, porous 3D scaffolds with cells and signaling factors to promote cell adhesion, proliferation, osteogenic differentiation, tissue function and vascularization.^19^ These scaffolds are designed to replicate the structure and function of natural bone ECM and are often combined with osteoprogenitor or differentiated bone cells.^3^ Common bioresorbable biomaterials include natural polymers (*e.g.* collagen, gelatin, silk fibroin, and chitosan), synthetic polymers (*e.g.* polylactic acid (PLA), polyglycolic acid (PGA), and polycaprolactone (PCL)) and ceramics (*e.g.* HAp, beta-tricalcium phosphate (β-TCP), and bioactive glasses (BGs)).^17^ Each material has distinct advantages and limitations: natural polymers offer biocompatibility and biological activity but have weak mechanical properties, while synthetic materials provide robust mechanical strength, tunable physical properties and processability but lack bioactivity.^20^ Composite scaffolds, which integrate synthetic and natural materials, have demonstrated improved performance by providing the bioactivity, mechanical strength and structural integrity required for BTE.^21^ Nonetheless, recreating the intricate bone microenvironment remains challenging using conventional chemical and physical methods, due to the ECM's complex composition and structure. Furthermore, most strategies employ single proteins to mimic the highly complex bone ECM microenvironment, and they often present limited outcomes.^22^ A notable emerging approach in the tissue engineering (TE) field is the use of decellularized ECM (dECM), which is derived from the decellularization of native tissues and organs or cultured cells.^23^ dECM preserves biochemical cues essential for cell adhesion, proliferation and differentiation, thereby closely mimicking the native tissue microenvironment and outperforming single-component ECM mimetics. Among various cell sources, MSCs have been widely used to produce dECM for a variety of TE applications, including bone,^24^ cartilage,^25^ and adipose tissue,^26^ given their ability to differentiate into various cell types, their ease of *in vitro* expansion, their availability from multiple tissue sources and their advantageous secretory profile of bioactive paracrine factors.^27^

In particular, the integration of MSC-derived dECM with synthetic polymers has enabled the development of composite scaffolds that meet both the biological and mechanical requirements for bone repair. These hybrid constructs offer superior bioactivity compared with purely synthetic scaffolds, reflecting a pivotal shift in BTE.^28,29^ Current research suggests that effective bone regeneration depends not just on mimicking structural features, but, more importantly, on delivering dynamic biochemical cues that guide cellular behavior, immune response, and tissue remodeling.^30^ As such, there is an increasing emphasis on bottom-up, bioactivity-driven strategies that move beyond traditional top-down approaches centered on replicating bones’ structural and mechanical features. These innovative approaches leverage the regenerative potential of biologically instructive matrices, such as MSC-derived dECM, which provide essential bioactive cues for bone repair.^31^

While several reviews have examined the use of synthetic scaffolds in BTE, most have focused on material selection,^32,33^ scaffold design parameters^32,34^ and fabrication methods.^33^ Likewise, the decellularization, characterization and application of tissue- and cell-derived dECM have been reviewed across various TE contexts.^35^ However, none of these works have specifically focused on MSC-derived dECM or its integration with synthetic scaffolds for BTE. This review addresses this gap by exploring how MSC-derived dECM can be harnessed to enhance the bioactivity of synthetic scaffolds. We explore the key strategies used in this approach, namely the incorporation of dECM into prefabricated scaffolds and the *in situ* decoration of scaffolds with dECM, and evaluate their effects on osteogenic differentiation. Additionally, we highlight current challenges and outline future research directions essential for promoting the application of such scaffolds in bone regenerative medicine.

## 2. Cell-derived dECM *vs.* tissue-derived dECM

dECM can be obtained from tissues/organs (tissue- or organ-derived dECM) or from cultured cells (cell-derived dECM).^36^ Tissue-derived dECM preserves the native 3D architecture and microstructure of the original tissue, offering adequate mechanical support, which is important for TE applications. Notably, a variety of tissues, including bone, skin, urinary bladder matrix, blood vessels and heart valves, have been successfully decellularized while maintaining their structural integrity, enabling their use in preclinical research and clinical therapies.^37–41^ Specifically, for BTE, bone tissue has been demineralized and decellularized to produce bone tissue-derived dECM, which retains the osteoconductive properties of native bone and has been used to produce 3D bioprinted and biomimetic bone scaffolds.^42,43^ Xenogeneic tissues, sourced from animals, are a common source for tissue-derived dECM due to their high availability.^44^ However, the resulting dECM faces challenges such as the risk of eliciting immune responses or graft rejection caused by residual immunogenic materials, and the risk of pathogen transmission.^36^ To mitigate these risks, harsh decellularization treatments are often employed, which can inadvertently remove key bioactive ECM components critical for cell signalling.^45^ Alternatively, allogeneic tissues, sourced from living donors or cadavers, offer improved biocompatibility and reduced immunogenicity but, nonetheless, also have limitations including limited availability of donor tissues and organs^46^ and the uncontrolled tissue variability that occurs due to the age, health and gender of the donor.^47^

Cell-derived dECM appears as an alternative to tissue-derived dECM, consisting of a complex and organized mixture of macromolecules that can mimic the native cell niche, which is obtained by decellularization of *in vitro* cell cultures. This approach benefits from greater availability of cell sources, higher reproducibility and the use of less aggressive decellularization protocols.^48^ Additionally, maintaining cell cultures under pathogen-free conditions reduces the risk of disease transmission often associated with donor tissues. Cell-derived dECM also offers greater customization, unlike tissue-derived dECM, as its composition can be tailored by selecting specific cell types, culture systems (2D *vs.* 3D, monocultures *vs.* co-cultures, static *vs.* perfusion systems)^49^ and applying targeted stimuli to enhance or regulate ECM production.^50^ Furthermore, genetic modifications of the cell sources can enhance or supress the expression of specific ECM components.^51^ Nonetheless, cell-derived dECM often results in lower yields and lacks the intricate hierarchical structure and biomechanical strength inherent to native tissue-derived matrices,^52^ which may restrict its utility in applications requiring robust structural or biomechanical properties such as BTE.

## 3. Decellularization treatments

Decellularization refers to the process of removing cellular components from tissues, organs or cell cultures, while preserving the ECM architecture and composition.^53^ Ideally, the resulting dECM retains the structural, biochemical and biomechanical properties of the native ECM,^36^ serving as a scaffold for recellularization or as a material for post-processing fabrication techniques such as hydrogel formulation, 3D bioprinting and electrospinning.^54,55^

Decellularization techniques are classified into physical, chemical and enzymatic methods. These different methods influence the structure and composition of the dECM in distinct ways and have different advantages and limitations, as summarized in Table 1. Therefore, selecting the appropriate method, agent type, concentration (where applicable) and exposure time is crucial to maximize the removal of nuclear material while minimizing the loss of native ECM components.^48^ These choices depend on the specific characteristics of the material being decellularized and the intended application of the dECM.^55^ For instance, tissues and organs generally require more time and complex protocols to achieve complete decellularization, whereas cell cultures can be decellularized more rapidly using simpler methods.^56^ Additionally, whole organs with intact vascular networks typically require more aggressive decellularization strategies compared with thin, avascular tissues, due to their structural complexity.

**Table 1:** Advantages and limitations of the most used decellularization methods

Decellularization methods	Target source	Advantages	Limitations	References
Chemical				36, 48, 49, 54, 55 and 78–81
Acids (*e.g.* peracetic acid)	Cultured cells, tissues and organs	Effective cell removal and pathogen inactivation	Potential ECM protein and structural instability	
Nonionic detergents (*e.g.* Triton X-100)		Gentle on ECM; preservation of structural proteins and enzymatic activity; effective with enzymes	Long treatment times and multiple cycles are often required; potential loss of GAGs and other ECM components	
Ionic detergents (*e.g.* SDS)		Highly effective cell removal	More aggressive; potential ECM damage and residual cytotoxicity	
Zwitterionic detergents (*e.g.* CHAPs)		Effective cell removal; preservation of ECM ultrastructure	Incomplete decellularization; presence of cellular debris; ECM destruction at high concentrations	
Hypotonic solutions (*e.g.* Tris–HCL)		Dissociation of cellular membranes without harsh chemicals	Incomplete decellularization when used alone	
Hypertonic solutions (*e.g.* sodium chloride)		Effective cell lysis	Often requires combination with other agents	
Bases (*e.g.* ammonium hydroxide)		Effective cell removal and pathogen inactivation	Potential ECM damage	
Enzymes				
Trypsin	Cultured cells, tissues and organs	Effective removal of cellular debris	ECM protein degradation with overuse	
Nucleases (*e.g.* DNAse, RNase)		Efficient DNA removal; reduced immunogenicity	May require combination with detergents; prolonged exposure can alter ECM structure and weaken mechanical integrity; additional cleaning steps required	
Physical				
Agitation and immersion	Tissues and organs	Simple technique; cost-effective; suitable for smaller tissues without blood vessels	Incomplete decellularization without chemicals; large quantities of decellularization agents required	
Sonication		Reduced time; enhanced cell removal	Potential ECM damage with protein denaturation	
Freeze-thawing		Effective cell lysis; preservation of ECM mechanical properties	Multiple cycles required; potential residual DNA fragments; potential ECM damage if temperature is not carefully controlled	
Electroporation		Effective cell removal with minimal chemicals	Need for specialized equipment; high cost	
Supercritical carbon dioxide		Efficient; preservation of ECM and mechanical properties	Need for specialized equipment and precise control	
Perfusion		Uniform decellularization; suitable for large tissues	Need for specialized equipment and optimization; time-consuming	

Physical decellularization techniques are predominantly used for tissues and organs and rely on physical stress to disrupt cell membranes and lyse cells without significantly affecting the tissue's structure.^57^ Common methods include freeze-thawing, sonication and electroporation. Freeze-thaw cycles promote the formation of ice crystals that puncture cell membranes; sonication applies mechanical pressure to disrupt cells, and electroporation delivers electrical pulses to create nanopores in cell membranes, resulting in cell death.^36^ Mechanical force has also been used to remove the cell layers of tissues (*e.g.*, small intestine and the urinary bladder).^58^ More recently, supercritical CO_2_ has emerged as another effective physical decellularization method.^54^

Although physical methods are generally less damaging to the ECM structure, these are often insufficient on their own and are therefore combined with chemical or enzymatic approaches to achieve complete decellularization.^59^ For larger tissues and vascularized organs, perfusion decellularization, which involves the perfusion of chemical and/or enzymatic agents through the tissue's vascular network, is frequently employed – this ensures uniform decellularization and preservation of the entire macrostructure of the organ.^60^ Conversely, for smaller avascular tissues, agitation and immersion decellularization is preferred, whereby they are fully immersed in decellularization solutions, sometimes after mincing or slicing the tissue to increase surface area. While this approach is faster and more efficient for smaller samples, it requires larger quantities of decellularization agents and may compromise the ECM ultrastructure.^61^

Chemical treatments involve agents that target intercellular connections and degrade cellular components.^36,56^ Detergents, such as Triton-X100 (nonionic), sodium dodecyl sulfate (SDS, ionic) and 3-(3-cholamidopropyl) dimethylammonio-1-propanesulfonate (CHAPs, zwitterionic) are commonly used to solubilize cell membranes and degrade DNA.^48^ Acid and bases, such as peracetic acid or ammonium hydroxide,^54,56^ disrupt cellular components through pH-driven mechanisms, while hypo- and hypertonic solutions create osmotic stress that lead to cell lysis and detachment.^62^

Enzymatic decellularization methods utilize enzymes to degrade cellular components, with trypsin, which cleaves peptide bonds, and nucleases, which degrade nucleic acids, being the most commonly used.^27^ However, enzymatic treatments are typically combined with chemical agents to improve their efficacy, as enzymes alone often cannot achieve complete decellularization.^56^ Moreover, enzyme residues in the dECM may hinder recellularization, commonly requiring additional washing steps.^63^

The success of a decellularization method is typically assessed by the residual DNA content and the structural preservation of the ECM. Residual DNA levels should be less than 50 ng per mg of dry ECM and/or DNA fragments should be less than 200 bp in length, with no visible nuclei remaining.^54^ Additional assessments, such as collagen and glycosaminoglycan (GAG) quantification and biomechanical analysis, are recommended to confirm that the dECM retains the structural and functional properties of the native material.^64^

Following decellularization, both tissue/organ- and cell-derived dECM can be used in either non-solubilized or solubilized forms, depending on the application. Non-solubilized tissue-derived dECM retains the native 3D structure and mechanical properties of the source tissue, and is often used as a whole scaffold in top-down TE approaches.^65,66^ However, the benefits of retaining native tissue architecture in BTE remains debated, with some studies suggesting limited advantages – particularly given that scaffold geometry and porosity can be finely controlled using advanced fabrication techniques such as 3D printing.^67,68^ In contrast, non-solubilized cell-derived dECM is typically employed as cell sheets^69^ or as a decorative coating on prefabricated scaffolds,^70^ providing a biologically active surface without disrupting ECM structure.

Solubilized dECM, whether tissue- or cell-derived, is obtained *via* enzymatic digestion (*e.g.*, pepsin) or chemical extraction (*e.g.* with urea), which disrupt the native matrix organization.^71^ Despite this loss of structural integrity, solubilized dECM is widely used in bottom-up approaches because it offers greater versatility in processing, being easily incorporated into hydrogels, bioinks or electrospun fibers.^72–76^ Moreover, solubilization may improve the bioavailability of ECM components, promoting improved cell–matrix interactions.^77^

## 4. MSC-derived dECM

MSCs are multipotent, non-hematopoietic cells that can be isolated from various tissues, including bone marrow, adipose tissue, dermis, dental pulp, synovial membrane, peripheral blood, and umbilical cord and placental blood.^82–88^ These cells can self-renew and differentiate into multiple tissue types such as bone, cartilage and fat,^89,90^ making them key players in tissue repair and regeneration. Beyond their differentiation potential, MSCs also secrete a wide range of paracrine factors, such as growth factors, ECM components and extracellular vesicles, that work synergistically to modulate inflammation, promote angiogenesis, prevent apoptosis, and stimulate the survival, proliferation and differentiation of resident tissue-specific cells.^91^ These regenerative, immunomodulatory and trophic properties render MSCs highly valuable for regenerative medicine and the treatment of degenerative diseases. Despite being a promising approach, direct transplantation of MSCs to injury sites or systemic circulation faces important challenges such as MSC entrapment in non-targeted organs and risk of embolization.^89^ Consequently, there has been an increasing interest in using MSC-derived products, particularly their paracrine factors and ECM, as alternative strategies for TE applications.

MSC-dECM is a natural material with excellent biocompatibility and bioactivity. It acts as a reservoir of cytokines and growth factors that regulate key biological processes such as inflammation (*e.g.*, MCP-1, M-CSF, IL-8), angiogenesis (*e.g.*, VEGF-A), and tissue remodelling (*e.g.*, MMP-13, OPG).^92,93^ This composition makes MSC-dECM an appealing option for regenerative applications across several tissue types. Indeed, MSC-dECM has been shown to promote MSC proliferation,^94^ rejuvenate aged mouse stem cells,^95^ enhance lineage-specific differentiation,^96^ and support chondrocytes proliferation while preserving their phenotype.^97^ In wound healing, MSC-dECM facilitates matrix deposition and cell adhesion through the high expression of fibronectin and extracellular matrix protein-2.^98^ Additionally, MSC-dECM offers protection against oxidative stress, as MSCs cultured on MSC-dECM exhibit expression of antioxidative enzymes^99^ and lower reactive oxygen species (ROS) production.^94,95,99^

The composition of MSC-dECM is influenced by the cell source and culture conditions used for its generation.^51^ Notably, when MSCs are cultured under osteogenic conditions, they secrete an ECM enriched with bone-specific biochemical and osteoinductive cues.^24,100^ Upon decellularization, this dECM retains key structural proteins and bioactive molecules that, when reseeded with new cells, help direct their osteogenic differentiation – making MSC-dECM highly suitable for BTE strategies. Among MSC sources, bone marrow-derived MSCs (BMSCs) are particularly effective for bone regeneration due to the osteoinductive profile of their secreted ECM, which closely mimics native bone marrow ECM.^101,102^ Hoch *et al.*^103^ demonstrated that BMSCs reseeded onto BMSC-dECM exhibited improved proliferation, osteogenic differentiation and proangiogenic factor secretion, even in the absence of osteogenic media. Moreover, the transplantation of cells combined with BMSC-dECM improved bone formation, vessel density and cell viability.^103^ BMSC-dECM has also been shown to inhibit osteoclastogenesis by attenuating ROS^104^ and to enhance the osteogenic potential of adipose-derived MSCs.^105^

Given these advantages, different techniques have been used to stimulate the production of ECM components by MSCs,^106^ ranging from the traditional 2D monolayers^107^ to more advanced biomimetic strategies, such as 3D culture systems^108^ and bioreactors.^109^

## 5. MSC-dECM enhancing the biological performance of synthetic scaffolds for BTE

**Fig. 1:**
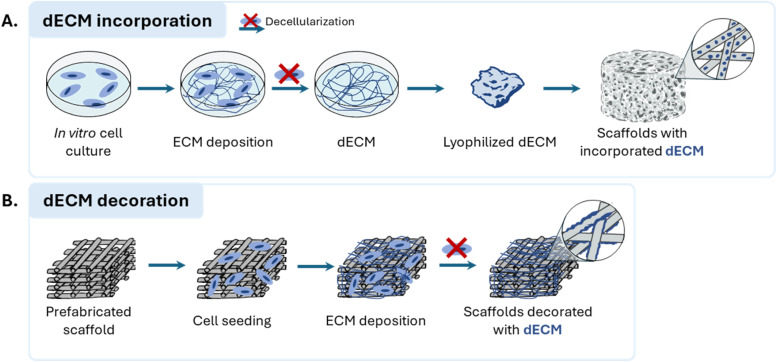
Strategies reported in the literature for combining MSC-dECM with synthetic scaffolds for bone regeneration applications, namely (A) dECM incorporation into biomaterial solutions and (B) dECM *in situ* decoration of prefabricated scaffolds.

MSC-dECM has been combined with synthetic scaffolds to enhance their bioactivity through two primary strategies: incorporation into biomaterial solutions and *in situ* scaffold decoration. In the first approach, dECM is produced *in vitro*, typically lyophilized into a powder and/or solubilized *via* pepsin digestion, and subsequently incorporated into biomaterial solutions. These formulations can then be used to create hydrogels or applied in electrospinning and 3D (bio)printing. Alternatively, MSCs can be seeded directly onto pre-fabricated scaffolds, where they secrete ECM during *in vitro* culture. Following decellularization, the resulting scaffolds are coated with non-solubilized dECM that conforms to the existing scaffold geometry, enabling the recreation of biomimetic cues and complex ECM patterns.^28,110^ These strategies are illustrated on Fig. 1.

Although solubilized dECM is widely used in TE, particularly in the formulation of bioinks for 3D bioprinting,^37,111,112^ its use in the context of MSC-dECM integrated into synthetic scaffolds for BTE remains limited. Instead, most MSC-dECM composite scaffolds have been developed using the dECM decoration approach, where dECM is used in its native, non-solubilized form. This preference likely stems from concerns that solubilization, particularly *via* pepsin digestion, may disrupt the nanoarchitecture and functional integrity of the dECM, despite the retention of many bioactive cues.^28^*In situ* dECM decoration avoids these issues, however, often results in uneven ECM distribution on the scaffold due to nutrient and oxygen gradients in static cultures. While perfusion bioreactors can mitigate these nutrient limitations, they introduce fluid-flow-induced variable shear stress, which may still lead to uneven ECM deposition.^113^ To address these challenges, a more uniform incorporation of dECM into scaffolds can be achieved by solubilizing the dECM and thoroughly mixing it with other biomaterial solutions prior to scaffold fabrication. Additionally, dECM lyophilization, performed before solubilization, facilitates water removal and stabilizes ECM proteins, thereby improving the retention of their bioactivity.^114^

In bone regeneration strategies, common synthetic materials used in combination with MSC-dECM include PCL, TCP, BCP, and titanium (Ti), all of which offer tunable mechanical properties and structural support, making them highly promising for BTE applications.

### 5.1 3D-printed scaffolds

The combination of MSC-dECM with 3D printed synthetic scaffolds has been explored primarily through *in situ* dECM decoration, often applied in PCL scaffolds.

**Fig. 2:**
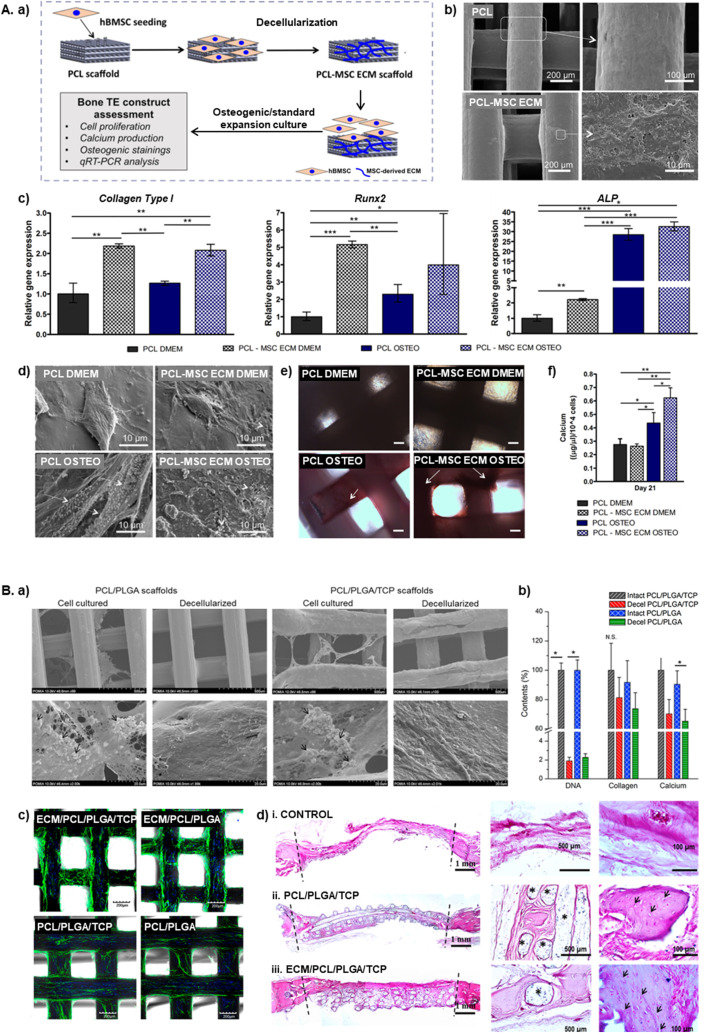
3D-printed scaffolds decorated with MSC-dECM for enhanced bone regeneration. (A) PCL-MSC ECM scaffolds developed by Silva *et al.*^115^ to better mimic the native bone niche. (a) Schematic of the experimental plan for generating PCL-MSC ECM scaffolds and evaluating their ability to promote MSC proliferation and osteogenic differentiation. (b) SEM images showing the morphology of pristine PCL (top) and PCL-MSC ECM (bottom) scaffolds, confirming the presence of MSC-ECM in the latter. (c) Expression levels of osteogenic marker genes (*Collagen Type I*, *Runx2* and *ALP*) after 21 days of MSC culture on the scaffolds under osteogenic and standard expansion medium. (d) SEM images of MSCs cultured on the scaffolds for 21 days under osteogenic and standard expansion media. White arrows denote mineralized nodules. (e) ALP/Von Kossa stainings of MSCs cultured for 21 days under osteogenic differentiation and standard expansion medium. Arrows denote calcium deposits. Scale bar: 100 μm. (f) Quantification of calcium deposition by MSCs seeded on the scaffolds after 14 and 21 days under osteogenic differentiation medium and standard expansion medium (*p < 0.05, **p < 0.01, ***p < 0.001). Adapted from ref. 115 with permission from John Wiley and Sons, copyright 2020. (B) PCL/PLGA/β-TCP scaffolds ornamented with mineralized ECM deposited by hMSCs, designed by Pati *et al.*^117^ (a) SEM images of the scaffolds before and after decellularization. Black arrows indicate cell-laid ECM. (b) DNA, collagen, and calcium content of the scaffolds before and after decellularization (*p < 0.05; N.S., not significant) (c) *F*-actin staining of hMSCs on the different scaffold types after 24 h. (d) Hematoxylin&Eosin stainings of calvaria defects treated with (i) control, (ii) PCL/PLGA/TCP scaffolds and (iii) ECM/PCL/PLGA/TCP scaffolds. Dotted lines outline the defect area. In the control group (i), the defect margins are connected by a thin, dense connective fibrous tissue; defects treated with PCL/PLGA/TCP scaffolds (ii) are largely occupied by dense fibrous tissue, whereas those treated with ECM/PCL/PLGA/TCP scaffolds (iii) are nearly filled with bone-like tissue (* indicates remaining scaffold fragments). Adapted from ref. 117 with permission from Elsevier, copyright 2015.

Silva *et al.*^115^ investigated the effect of MSC-dECM *in situ* decoration on 3D printed PCL scaffolds fabricated *via* fused deposition modelling (FDM), which exhibited high porosity and interconnectivity (Fig. 2A). After MSC-dECM decoration, fibronectin and laminin were detected on the scaffolds' surface, although not uniformly distributed. dECM-coated scaffolds enhanced MSC attachment and proliferation, as well as expression of *RUNX2* and *COL1A1*, without osteogenic supplementation, compared to pristine PCL scaffolds, which could be explained by the presence of cytokines within or recruited by the deposited scaffolds.^115^ These findings align with the work of Deutsch *et al.*,^116^ who also used FDM-printed PCL scaffolds, but incorporated collagen type I as a coating before MSCs or amniotic fluid stem cells (AFSCs) were seeded for ECM production under dynamic flow conditions. After decellularization, MSCs were cultured with media supplemented with β-glycerol phosphate and ascorbic acid, producing mineralized matrix at a similar rate as MSCs seeded in non-coated scaffolds cultured with osteogenic media, demonstrating that MSC-dECM alone was sufficient to drive osteogenic differentiation. Interestingly, AFSC-derived dECM induced even greater production of mineralized matrix, suggesting that the cell source of ECM plays a crucial role in determining its osteogenic potential. Additionally, the dECM-coated scaffolds contributed to bone healing *in vivo*, with improved rate of bridging, compared with non-coated scaffolds.^116^

Building upon the osteogenic potential of MSC-dECM in PCL scaffolds, Pati *et al.*^117^ incorporated MSC-dECM into a more complex 3D-printed scaffold composed of PCL, PLGA and β-TCP (Fig. 2B). ECM deposition was performed under dynamic culture in a rotary flask bioreactor with osteogenic media, to promote the formation of mineralized ECM. The ECM-decorated scaffolds supported upregulation of *RUNX2*, *ALP*, *OC* and *OPN* genes, and exhibited increased calcium deposition compared to bare 3D-printed scaffolds. Additionally, the scaffolds showed superior *in vivo* performance in ectopic and orthotopic rat bone models, promoting greater host cell invasion and bone mineralization, compared with bare scaffolds, demonstrating their enhanced osteoconductive and osteoinductive properties.^117^

In a similar approach, Tan *et al.*^118^ explored the effects of MSC-dECM in 3D printed scaffolds composed of gelatin, alginate, and synthetic bioactive glass, which is a bioceramic highly interesting in BTE as it can quickly dissolve into osteostimulative ions that drive tissue mineralization.^119^ Endothelial cells (EC)-dECM was also used to decorate the scaffolds. While both dECM types enhanced the expression of *RUNX2* and *BMP-2* genes compared to pure scaffolds, EC-dECM scaffolds exhibited even higher osteogenic marker expression, along with angiogenic genes upregulation. *In vivo*, both ECM-decorated scaffolds promoted bone healing, with partial degradation of the scaffold material and extensive new bone formation. The additional angiogenic effects of EC-dECM suggest that incorporating ECM from multiple cell types could further enhance vascularization in bone regeneration applications.^118^

### 5.2 Micro-/nano-electrospun fibrous scaffolds

**Fig. 3:**
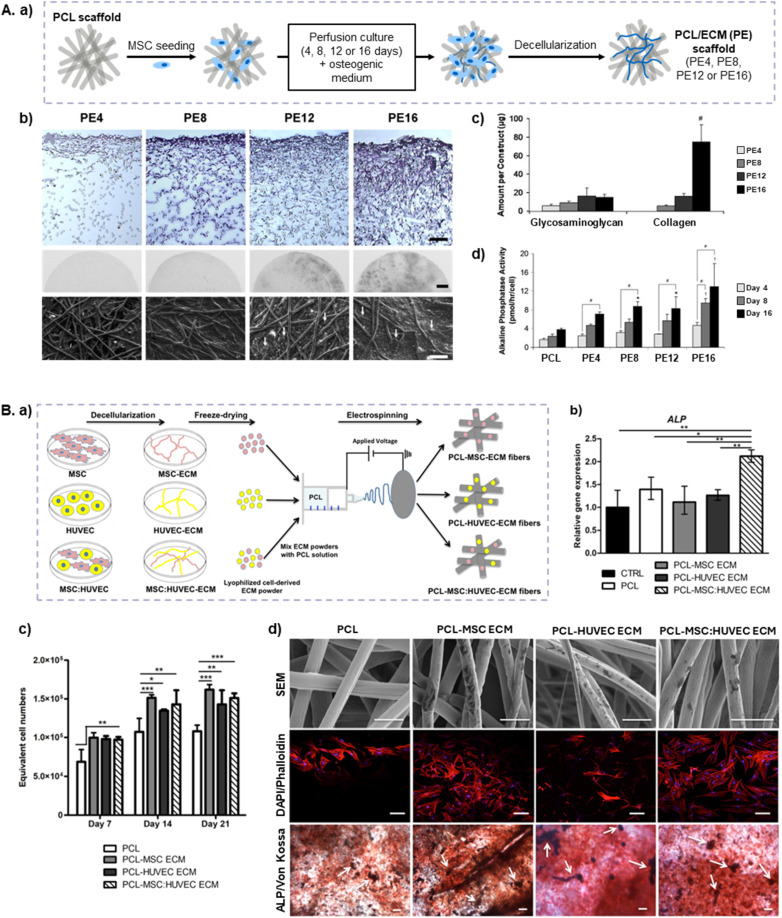
Electrospun scaffolds developed using MSC-dECM for bone regeneration. (A) PCL/ECM (PE) electrospun constructs developed by Liao *et al.*^122^ under perfusion with dexamethasone. (a) Schematic of the production of PE constructs with increasing culture durations (4, 8, 12, and 16 days), resulting in scaffolds decorated with mineralized matrix of various maturities (PE4, PE8, PE12, and PE16). (b) Scaffold matrix evaluation using Hematoxylin&Eosin staining (top; scale bar: 100 μm), X-ray imaging (middle; scale bar: 1 mm) and SEM (bottom; scale bar: 100 μm). Arrows indicate mineral nodules. (c) Glycosaminoglycan and collagen contents of the PE constructs. (d) ALP activity of PCL and PE scaffolds seeded with MSCs and cultured without dexamethasone for 16 days (#, *, †*p* < 0.05; #, *, † indicate a significant difference in expression level between time points, *vs.* PCL and *vs.* all other scaffold groups, respectively). Adapted from ref. 122 with permission from Elsevier, copyright 2010. (B) 3D microporous electrospun dECM/PCL scaffolds produced by Carvalho & Silva *et al.*^124^ (a) Schematic of the experimental procedure, depicting electrospinning of dECM-enriched PCL solutions. (b) ALP expression by MSCs seeded on the scaffolds. (c) MSCs proliferation on the scaffolds at day 21. (d) SEM micrographs (top; scale bar: 5 μm), DAPI/Phalloidin stainings (middle; scale bar: 100 μm) and ALP/Von Kossa stainings (bottom; scale bar: 200 μm) of the scaffolds, demonstrating ALP activity of MSCs cultured on the scaffolds (reddish areas) (*p < 0.05, **p < 0.01, ***p < 0.001). Adapted from ref. 124 with permission from Elsevier, copyright 2019.

Electrospinning is a widely used technique to fabricate fibrous and porous scaffolds that mimic the hierarchical micro/nano scale fibrous structure of native bone ECM.^120^ Several studies have explored the integration of MSC-dECM with electrospun PCL scaffolds to enhance their osteogenic potential. Thibault *et al.*^121^ demonstrated that prior exposure to dexamethasone during MSC-ECM deposition on PCL scaffolds initiates osteogenesis, while subsequent culture in ECM-decorated constructs sustains osteogenic differentiation, including ALP activity and calcium production, without additional osteoinductive cues. Similar findings were reported by Liao and colleagues,^122^ who also developed MSC-dECM-decorated PCL electrospun scaffolds, but under flow perfusion conditions with dexamethasone (Fig. 3A). MSCs penetrated the interconnected pores of the scaffold and differentiated along the osteogenic lineages. The composition of the mineralized dECM, namely in terms of mineral, collagen and GAGs, differed depending on the stage of osteogenesis. Among the different dECM-decorated scaffold types, those containing the most mature mineralized matrix exhibited the highest ALP activity and calcium content, reinforcing the hypothesis that ECM maturity influences osteogenic differentiation, even in dexamethasone-free conditions.^122^ Using a similar setup, Thibault *et al.*^123^ further investigated whether MSC-ECM deposited on electrospun PCL constructs under flow perfusion conditions replicated the protein and mineral composition of mature bone tissue. Using liquid chromatography tandem mass spectrometry (LC-MS/MS) analysis, the authors found that the dECM initially consisted of adhesion proteins such as fibronectin, essential for early bone formation. Over time, collagen type I, HAp, matrix remodelling proteins, and regulatory proteins, including MMP-2 and PEDF, accumulated in the dECM, indicating that prolonged culture enhanced the scaffold's bone regenerative potential through improved mineralization.^123^

Unlike these studies, which utilized *in situ* dECM decoration, Carvalho & Silva *et al.*^124^ employed the ECM-incorporating approach to fabricate dECM/PCL electrospun scaffolds (Fig. 3B). Lyophilized dECM powder, derived from MSC : HUVEC co-cultures, was blended into the PCL solution before electrospinning. The scaffolds had high porosity and interconnectivity, and the addition of dECM did not impact its mechanical properties, which were similar to those of demineralized human trabecular bone (tensile modulus ∼12 MPa). dECM incorporation significantly enhanced MSC proliferation compared to PCL scaffolds, while also increasing calcium deposition, ALP activity and expression of the osteogenic marker genes *RUNX2*, *ALP* and *OPN*. Notably, scaffolds containing MSC : HUVEC-derived dECM outperformed MSC- or HUVEC-only dECM scaffolds in promoting osteogenic differentiation, corroborating the potential benefits of endothelial–mesenchymal interactions in BTE strategies.^124^ In another study. Padalhin *et al.*^125^ co-cultured pre-osteoblasts (MC3T3-E1) and MSCs on PCL scaffolds, followed by decellularization and reseeding with pre-osteoblasts. The dECM-coated scaffolds exhibited improved *in vitro* cell proliferation and increased *ALP* and *OPN* gene expression when compared with uncoated scaffolds. Upon implantation in rat skull defects, acellular ECM-PCL scaffolds enhanced bone formation *via* endochondral ossification and exhibited minimal inflammation, likely due to the biocompatibility of PCL and the use of dECM derived from biologically related species (rat and mouse). Interestingly, PCL-dECM scaffolds also promoted cartilage-like nodule formation within the inter-fiber space, indicating that they function well as artificial callus, capable of supporting robust formation of intermediate bone tissue.^125^

### 5.3 Hydrogels and sponges

**Fig. 4:**
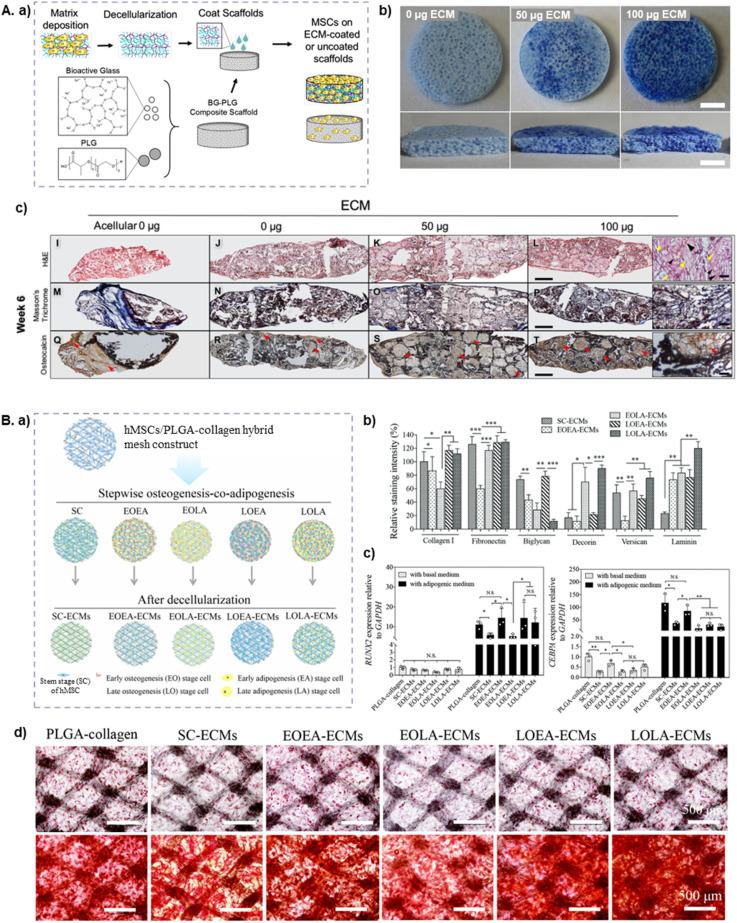
Hydrogel-based scaffolds functionalized with MSC-dECM for bone regeneration. (A) Microporous PLG/bioactive glass (BG) coated with MSC-dECM to improve MSC proliferation and survival, developed by Harvestine *et al.*^127^. (a) Schematic of the experimental design followed for the production of the scaffolds. (b) Coomassie Brilliant Blue staining of BG-PLG scaffolds: uncoated (left), coated with 50 μg of ECM (middle) and coated with 100 μg of ECM (right) (scale bar: 2 mm). (c) Histological analysis of explants at 6 weeks: Hematoxylin & Eosin staining (top), Masson's trichrome staining (middle) and osteocalcin (OC) immunostaining (bottom) (scale bar: 500 μm). Magnified views of the 100 μg ECM group are shown on the right (scale bar: 50 μm). Arrows denote blood vessels (black), connective tissue (yellow) and positive OC immunostaining (red). Adapted from ref. 127 with permission from American Chemical Society, copyright 2016. (B) PLGA/COL/dECM hydrogels designed by Chen *et al.*^128^ to study ECM remodeling during MSC osteogenesis and adipogenesis. (a) Schematic of the experimental workflow to prepare PLGA-collagen-ECMs hybrid meshes by decellularizing hMSCs/PLGA-collagen hybrid mesh constructs. hMSCs were controlled at different stages of osteogenesis-co-adipogenesis. (b) Quantification of ECM components (collagen I, fibronectin, biglycan, decorin, versican and laminin) normalized to the level of collagen I in the SC–ECM scaffold. (c) RT-PCR analysis of *RUNX2* (osteogenesis-related marker) and *CEBPA* (adipogenesis-related marker) in the scaffolds after culture in basal medium, osteogenic or adipogenic medium. (d) Oil Red O (top) and alizarin red S (bottom) stainings of the constructs after 14 and 21 days of culture, indicating lipid accumulation and calcium deposition in the scaffolds (*p < 0.05, **p < 0.01, ***p < 0.001). Adapted from ref. 128 with permission from Royal Society of Chemistry, copyright 2019.

Polyesterurethane (PEU) has been widely used in TE but, like most synthetic materials, it lacks tissue-specific bioactive cues. To address this, Sadr *et al.*^126^ used PEU foams seeded with BM-MSCs to explore bioreactor-based ECM production, creating off-the-shelf dECM-decorated polymeric hydrogels. By employing perfusion bioreactors, the authors achieved homogeneous cell seeding, improved cell viability, uniform ECM deposition and higher decellularization efficiency. The ECM-PEU scaffolds promoted osteogenic differentiation, *via* upregulation of bone sialoprotein (*BSP*), *OC* and *OPN* genes, along with increased calcium deposition, compared to bare PEU foams. *In vivo* implantation in mouse bone defects confirmed host cell invasion and the presence of co-localized mineral/BSP deposits that overlapped with loosely packed and organized ECM, resembling an early osteoid-like matrix.^126^ Similar results were obtained by Harvestine *et al.*,^127^ using microporous hydrogels composed of bioactive glass and poly (lactide-*co*-glycolide) (PLG) coated with MSC-dECM (Fig. 4A). Unlike the previous approaches, this coating was not done by *in situ* deposition but by treating the scaffold with solubilized dECM obtained after decellularization of 2D cultures. Without the presence of osteoinductive cues, the MSC-dECM coating increased the compressive modulus of the scaffolds (2.834 *vs.* 1.675 MPa), as well as MSC metabolic activity and proangiogenic VEGF secretion, while retaining OC expression, compared to uncoated scaffolds. Compared to uncoated scaffolds, *in vivo* implantation of dECM-coated scaffolds improved MSCs survival, tissue infiltration and osteogenic differentiation, assessed by OC staining.^127^ Building on these findings, Kim *et al.*^29^ developed porous scaffolds of BCP, which is an osteoconductive ceramic-coated synthetic polymer that integrates HAp and TCP. The scaffolds featuring interconnected pores (100–500 μm) were decorated with MSC-dECM, before being seeded and cultured with preosteoblasts (MC3T3-E1) under osteogenic conditions. Compared to bare BCP scaffolds, dECM decoration enhanced MC3T3-E1 cell attachment, proliferation and osteogenic differentiation, as evidenced by the upregulation of the bone-specific genes *OPN*, *ALP* and *BMP-2*.^29^

While the previous studies focused on scaffold-based bone regeneration, Chen *et al.*^128^ explored a different application within TE – the development of a 3D *in vitro* model to study dynamic ECM remodelling during MSC differentiation into osteogenic and adipogenic lineages (Fig. 4B). This is particularly relevant for osteoporosis research, given the characteristic imbalance between bone formation and fat accumulation in the bone marrow.^129^ The authors used microporous PLGA meshes hybridized with collagen microspheres to culture MSCs in different osteogenic and adipogenic media combinations, simulating the interplay between bone and fat tissue development.^128^ Their findings revealed that the dynamic composition of the dECM plays a crucial role in directing MSC differentiation into either bone or fat cells. Specifically, dECM from early osteogenesis/adipogenesis promoted adipogenesis while suppressing osteogenesis. In contrast, dECM derived from early osteogenesis and late adipogenesis suppressed both osteogenesis and adipogenesis, but enhanced MSC proliferation. Late-stage osteogenesis/adipogenesis ECM promoted osteogenesis with moderate effects on adipogenesis, whereas late osteogenesis and early adipogenesis ECM strongly favored the osteogenic differentiation of MSCs.^128^

### 5.4 Metallic scaffolds

Regarding metallic materials, titanium (Ti) is widely used to produce scaffolds for BTE applications, due to its excellent mechanical properties and high biocompatibility. Additionally, as shown in several studies, their bioactivity can be further enhanced through dECM functionalization.^130,131^ Pham *et al.*^132^ decorated Ti fiber meshes with MSC-dECM and observed that MSCs seeded on pure Ti scaffolds underwent osteogenic differentiation, but this was significantly enhanced when cells were cultured in decorated scaffolds, as evidenced by the upregulation of *ALP*, *OC*, osteomodulin (*OMD*), *OPN*, *RUNX2*, *BMP-3* and *FGF-2* gene expressions. This was accompanied by an increased deposition of mineralized matrix, indicating that dECM decoration promotes osteoinductive signalling on Ti scaffolds.^132^ Datta *et al.*^133^ conducted a similar study and further confirmed the increased osteogenic potential of MSC-dECM/Ti scaffolds. Even in the absence of osteogenic supplements, dECM-coated scaffolds exhibited higher calcium deposition and increased ALP activity compared to uncoated Ti scaffolds, highlighting the intrinsic osteoconductive properties of the pre-deposited dECM.^133^ Expanding on this, Datta *et al.*^134^ later introduced a flow perfusion system to improve ECM deposition within the scaffold porous structure. This dynamic culture method led to a 5-fold increase in calcium content compared to their previous static culture system.^133^ Compared to uncoated scaffolds, ECM-decorated Ti scaffolds exhibited a 40-fold increase in calcium content without dexamethasone supplementation and a 75-fold increase with dexamethasone, further supporting the role of dECM in enhancing osteogenesis. Additionally, ALP activity was significantly higher in Ti/ECM constructs. To investigate whether ECM-derived bioactive factors played a role in osteoinduction, Ti/ECM scaffolds were subjected to heat treatment to denature growth factors within the matrix. This resulted in a 60-fold decrease in calcium content, suggesting that the enhanced matrix mineralization observed in Ti/ECM scaffolds was primarily driven by the bioactive growth factors present in the pre-deposited matrix. These findings underscore the synergistic effect of dECM-derived bioactive cues and fluid-induced shear stresses in promoting MSC osteogenic differentiation, with significant implications for improving BTE applications.^134^

A summary of the MSC-dECM and synthetic polymer composite scaffolds developed for BTE applications can be seen in Table 2.

**Table 2:** Hybrid composite scaffolds for bone regeneration combining MSC-dECM and synthetic polymers in different configurations, including 3D printed scaffolds, electrospun fiber meshes, hydrogels/sponges and metallic scaffolds

Scaffold material	dECM strategy	Observation	Ref
**3D printed scaffolds**			
PCL	Decoration	MSC-dECM-decorated PCL scaffolds showed increased cell attachment and proliferation and enhanced expression of *RUNX2* and *COL1A1* without osteogenic media, compared with pristine PCL scaffolds	115
PCL	Decoration	Collagen-coated 3D printed PCL scaffolds were decorated with MSC-dECM produced *in situ*. MSCs cultured in the ECM/COL/PCL scaffolds under media supplemented with glycerol phosphate and ascorbic acid showed higher matrix production compared to MSCs cultured in COL/PCL scaffolds with osteogenic media. Bone healing was also improved in rat bone defects with the decorated scaffolds	116
PCL/PLGA/TCP	Decoration	MSC-ECM deposition in the scaffolds was stimulated using a bioreactor and osteogenic media. The presence of dECM improved further deposition of mineralized matrix by reseeded MSCs and enhanced osteogenic differentiation, by upregulating *RUNX2*, *ALP*, *OC* and *OPN* gene expression. *In vivo*, ECM-decorated scaffolds showed increased bone formation compared with bare scaffolds	117
Bioactive glass, gelatin, HAp	Decoration	The scaffolds were decorated with MSC- or endothelial cell (EC)-derived-dECM. Both scaffolds induced higher expression of osteogenic marker genes (*RUNX2*, *BMP-2*) compared with pure scaffolds. This was, however, increased in EC-ECM scaffolds. Both ECM-scaffolds promoted bone formation *in vivo*	118
**Micro-/nano-electrospun fibrous scaffolds**			
PCL	Decoration	The dECM-decorated scaffold supported MSC osteogenic differentiation, evaluated through ALP activity and calcium deposition, in the absence of dexamethasone supplementation	121
PCL	Decoration	Mineralized ECM was deposited by MSCs at various stages of ostegenesis in a flow perfusion bioreactor. The dECM-decorated scaffolds enhanced the osteogenic differentiation of MSCs, assessed by the higher ALP activity and calcium deposition, even in the absence of dexamethasone, especially those with the most mature matrices	122
PCL	Decoration	MSC-dECM deposition to the scaffold was promoted in a flow perfusion bioreactor. At later culture conditions, the dECM-PCL scaffold was composed of mature bone proteins, including COL I, HAp, matrix remodeling proteins and regulatory proteins	123
PCL	Incorporation	MSC : HUVEC dECM was added to the PCL casting solution and electrospun. The fibrous scaffolds promoted higher MSC proliferation, upregulation of *RUNX2*, *ALP* and *OPC* gene expression, and calcium deposition, when compared to PCL scaffolds	124
PCL	Decoration	MSCs and pre-osteoblasts were co-cultured on PCL electrospun fibers. The ECM-coated scaffolds showed improved *in vitro* cell proliferation and osteogenic differentiation, observed through increased *ALP* and *OPN* expression, and improved bone and cartilage nodules formation *in vivo*	125
**Hydrogels and sponges**			
PEU	Decoration	MSC-dECM deposition on the scaffolds was promoted using a perfusion system. The decorated scaffolds promoted upregulation of *BSP*, *OC* and *OPN* gene expression, and increased calcium deposition. *In vivo*, transplanted scaffolds showed invasion of host cells and the presence of co-localized minerals along BSP	126
Bioactive-glass/PLG scaffolds	Coating (solution)	MSC-dECM solution was used to coat the scaffolds, which promoted increased metabolic activity, decreased apoptosis and increased VEGF secretion by MSCs while retaining OC secretion, without osteogenic media, compared to non-coated scaffolds. Similar results were obtained *in vivo*	127
BCP	Decoration	MSC-dECM-decorated scaffolds, reseeded with preosteoblasts, showed improved cell proliferation, and osteoblast differentiation, as observed by the upregulation of *OPN*, *ALP* and *BMP-2* genes, when compared to plain scaffolds	29
PLGA/COL	Decoration	MSC-dECMs with osteogenic/adipogenic stage-specific compositions were deposited onto the scaffolds, influencing the proliferation and differentiation of MSCs	128
**Metallic scaffolds**			
Ti	Decoration	MSCs reseeded on ECM/Ti meshes showed upregulation of *ALP*, *OC*, *OMD*, *OPN*, *RUNX2*, *BMP-3* and *FGF-2* gene expression as well as an increased deposition of mineralized matrix	132
Ti	Decoration	MSCs reseeded on ECM/Ti meshes showed increased deposition of calcium and ALP activity even without osteogenic media, when compared to Ti meshes	133
Ti	Decoration	Mineralized MSC-dECM deposition on Ti scaffolds was enhanced in a flow perfusion system. Ti/ECM scaffolds showed increased calcium content and ALP activity compared with Ti and denatured Ti/ECM scaffolds, meaning that the positive effects on osteogenesis were due to the presence of growth factors in the deposited ECM	134

**Fig. 5:**
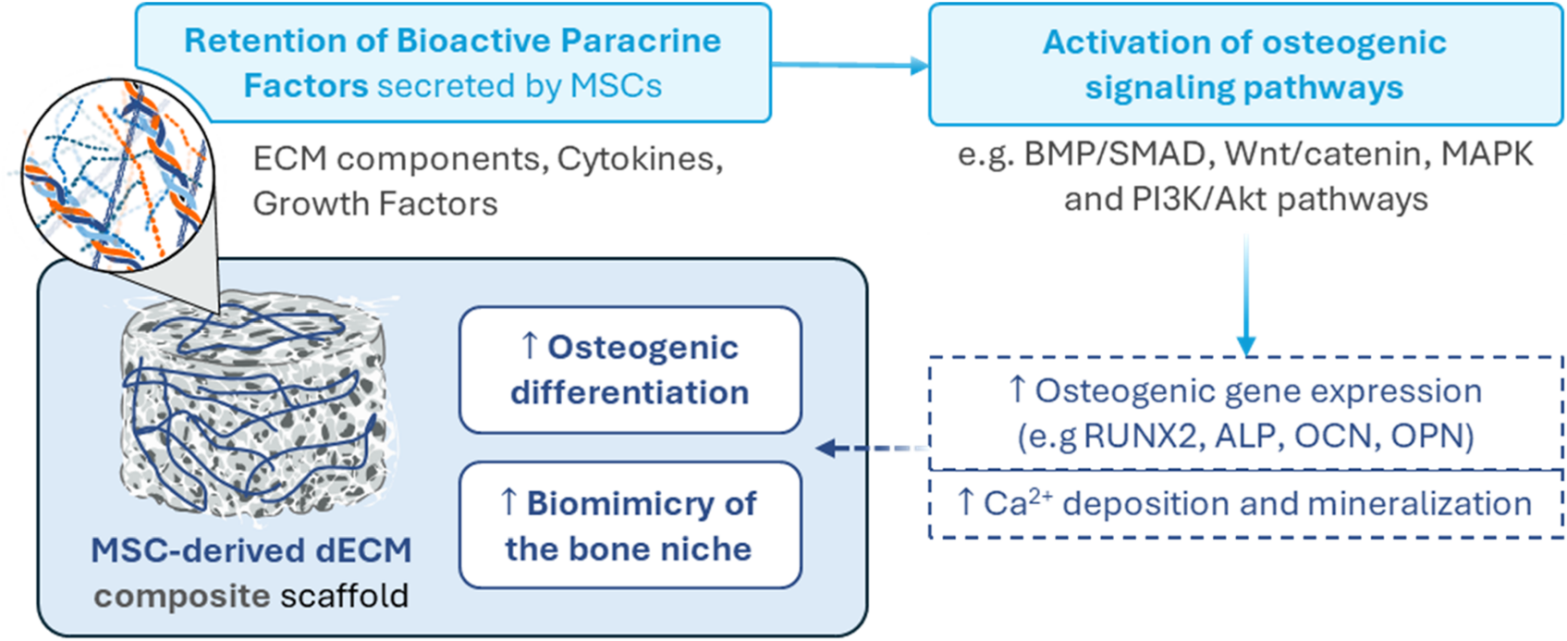
Schematic diagram illustrating how MSC-derived dECM composite scaffolds enhance osteogenic differentiation and mimic the native bone niche. The dECM provides a bioactive microenvironment that supports stem cell adhesion, proliferation, and upregulation of osteogenic gene expression, ultimately promoting mineralization and bone tissue formation.

Taken together, the studies reviewed in this chapter suggest that functionalizing synthetic scaffolds for BTE with MSC-derived dECM enhances their biological performance. This is evidenced by increased osteogenic differentiation, mineralization and *in vivo* bone formation (Fig. 5). However, most of these studies only compare MSC-dECM-enriched scaffolds with their unmodified counterparts. While such comparisons highlight the bioactivity improvements conferred by MSC-dECM, studies that evaluated other types of cell-derived dECMs, albeit limited in number, suggest that alternative dECM sources may perform equally well – or even better – in certain contexts. For instance, Tan *et al.*^118^ observed that EC-derived dECM triggered stronger osteogenic and angiogenic responses than MSC-dECM and Carvalho & Silva *et al.*^124^ reported superior outcomes using dECM from HUVEC-MSC co-cultures compared to MSC-only dECM.

Despite these observations, MSC-dECM remains widely used in BTE due to its ability to mimic aspects of the native bone microenvironment and retain key paracrine factors secreted by MSCs, such as VEGF and TGF-β, which are known to promote angiogenesis, modulate immune responses and recruit host progenitor cells. Nevertheless, to determine whether MSC-dECM is truly the most suitable choice for scaffold functionalization in BTE applications, future studies should assess how effectively these paracrine factors are preserved and remain bioactive within the scaffold environment and compare the functional outcomes of MSC-dECM with the ones achieved by other types of cell-derived dECMs.

## 6. Challenges and future trends

Cell-derived dECM is a versatile biomaterial with significant potential for TE applications. However, its transition into clinical practice remains challenging. Currently, only tissue- or organ-derived dECM has been successfully integrated into clinical practice, due to its superior mechanical properties and its ability to retain the micro- and macroarchitecture of the source tissue.^135^ Nevertheless, cell-derived dECM, including that derived from MSCs, offers advantages such as eliminating donor availability constraints, and its mechanical shortcomings can be addressed through combination with mechanically competent synthetic materials. Still, despite the promising benefits of MSC-dECM composite scaffolds for bone regeneration, several challenges must be addressed before they can be widely applied in clinical settings.

One major limitation of MSC-derived dECM and other cell-based dECMs is the low yield obtained during preparation, typically ranging from micrograms to grams per individual cultures.^136^ This presents difficulties in their integration into scaffolds, along with the unavoidable loss of bioactive factors during decellularization.^137,138^ To overcome these challenges, future research must focus on optimizing the decellularization process to minimize the loss of bioactive factors while simultaneously scaling up production. This involves creating ideal culture conditions that stimulate the desired ECM production, considering factors such as hypoxia, substrate stiffness and topography.^136^ One approach to scaling up MSC-derived dECM production is the use of culture platforms such as bioreactors and microcarriers, which have been reported to increase matrix deposition by MSCs.^139^ Additionally, the organization of MSCs into spheroids has been shown to significantly increase the expression and release of growth factors.^140^ Another promising strategy to enhance dECM yield is macromolecular crowding (MMC) using agents like Ficoll™, dextran sulfate, ascorbic acid, carrageenan, polyvinylpyrrolidone, and polyethene glycol.^141–143^ These macromolecules enhance matrix deposition by occupying space within the culture media, thereby concentrating essential molecules.^134,138^ MMC has been reported to enhance collagen type I deposition and alignment in MSC-dECM,^144^ as well as the presence of GAGs and growth factors such as FGF-2 and VEGF.^145^ Genetic modifications of cell sources, including overexpression of ECM proteins, activation of target pathways and silencing of metalloproteinases, represent additional strategies to enhance ECM production by MSCs.^52,136^

Another challenge in using MSC-derived dECM for BTE applications is the incomplete understanding of the mechanisms underlying its ability to promote bone tissue regeneration, particularly due to its complex composition. High-throughput proteomic analysis can provide detailed insights into dECM composition, which is crucial for ensuring scaffold functionality and biocompatibility.^138^ For instance, identifying and quantifying essential proteins involved in cell adhesion, migration, and tissue-specific differentiation can aid in optimizing MSC-derived dECM scaffolds for targeted clinical applications. Retention of these key proteins during decellularization would ensure that the scaffolds support the desired cellular behaviors.^54,146^ Finally, given the intrinsic variability in bone regeneration capacity among patients, future research should also explore the development of personalized scaffolds tailored to each patient's biological environment. This could be achieved by using patient-specific MSCs to generate autologous dECM, which can then be processed into a graft for implantation.^147^ Such personalized approaches would reduce the risk of immune rejection and improve integration with the host tissue.

## 7. Conclusions

MSC-dECM has emerged as a promising biomaterial for BTE applications, due to its bone-like composition and signaling, enhanced bioactivity, and osteogenic properties. Compared to tissue- and organ-derived dECM, MSC-dECM overcomes donor availability issues while still providing essential biochemical and biophysical cues that support bone regeneration. However, due to its poor mechanical properties, MSC-dECM has been combined with synthetic materials to create composite constructs that benefit from both enhanced structural integrity and osteoconductivity. This review highlighted the potential of integrating MSC-dECM with synthetic materials for bone regeneration across various scaffold systems, including 3D printed scaffolds, electrospun fibers, hydrogels, and metallic implant scaffolds. Two main approaches have been explored to generate MSC-dECM-enriched scaffolds, namely scaffold *in situ* decoration and dECM incorporation into biomaterial solutions. Both strategies have been shown to enhance the osteoconductive and osteoinductive properties of the scaffolds, effectively promoting osteogenic differentiation and bone formation *in vitro* and *in vivo*. Despite these promising findings, future research is needed to scale up MSC-dECM production, deepen our understanding of its regenerative potential and, ultimately, develop patient-specific scaffolds tailored to individual biological microenvironments. Implementing these strategies will bring us closer to translating MSC-derived dECM-based scaffolds into clinical practice, where they can serve as a viable alternative to autologous bone grafts.

## Conflicts of interest

The authors declare that they do not have any competing financial interest or personal relationships that could have appeared to influence the work reported in this paper.

## Data Availability

No primary research results, software or code have been included, and no new data were generated or analyzed as part of this review.
